# Bioinformatics Analysis Identifies Potential Ferroptosis Key Genes in the Pathogenesis of Intracerebral Hemorrhage

**DOI:** 10.3389/fnins.2021.661663

**Published:** 2021-06-07

**Authors:** Tongye Liu, Xinhe Li, Yiteng Cui, Pingping Meng, Guanghui Zeng, Qiang Wang, Yuyang Wang

**Affiliations:** ^1^Department of Rehabilitation Medicine, The Affiliated Hospital of Qingdao University, Qingdao, China; ^2^Department of Sports Medicine, Qingdao University Medical College, Qingdao, China

**Keywords:** intracerebral hemorhage, ferroptosis, TNF signaling pathway, *MAPK1*, bioinformactics

## Abstract

Intracerebral hemorrhage (ICH) is a dangerous neurological disease. The mechanism of ferroptosis in ICH remains unclear. Using bioinformatics analysis, we aimed to identify the key molecules involved in ferroptosis and provide treatment targets for ICH to further explore the mechanism of ferroptosis in ICH. GSE24265 was downloaded from the Gene Expression Omnibus (GEO) dataset and intersected with ferroptosis genes. A total of 45 differentially expressed genes (DEGs) were selected, most of which were involved in the TNF signaling pathway and oxidative stress response. Key modules constructed by the protein–protein interaction (PPI) network analysis and screening of genes related to the TNF signaling pathway led to the confirmation of the following genes of interest: *MAPK1*, *MAPK8*, *TNFAIP3*, *ATF4*, and *SLC2A1*. Moreover, *MAPK1* was one of the key genes related to TNF signaling and oxidative stress, and it may play an important role in ferroptosis after cerebral hemorrhage. The *MAPK1*-related molecules included *hsa-miR-15b-5P*, *hsa-miR-93-5P*, *miR-20b-5p*, *SNHG16*, *XIST*, *AC084219.4*, *RP11-379K17.11*, *CTC-444N24.11*, *GS1-358P8.4*, *CTB-89H12.4*, *RP4-773N10.5*, and *FGD5-AS1.* We also generated a hemorrhage rat model, which was used to conduct exercise intervention in ICH rats, and qRT-PCR was used to assess the expression levels of our genes of interest. The mRNA levels after cerebral hemorrhage showed that *MAPK1*, *ATF4*, *SLC2A1*, and *TNFAIP3* were upregulated, whereas *MAPK8* was downregulated. Treadmill training increased the expression of anti-inflammatory molecules *TNFAIP3* and *SLC2A1* and reduced the expression of *MAPK1, ATF4*, and *MAPK8*, indicating that treadmill training may be utilized as antioxidant therapy to decrease neuronal ferroptosis. The results of this study indicated that the *MAPK1*-related mRNA–miRNA–lncRNA interaction chain could be potentially employed as a biomarker of the inception and progression of ferroptosis after cerebral hemorrhage.

## Introduction

Intracerebral hemorrhage (ICH) is a disease associated with high mortality, disability, and dysfunction (Qureshi et al., 2001). The pathological accumulation of blood in the brain parenchyma occurs due to a sudden rupture of a blood vessel. Brain damage after ICH develops due to the growth of hematoma, which leads to the destruction of brain tissue and mechanical deformation (Xi et al., 2006). In addition, brain damage can occur due to mitochondrial dysfunction, microglia activation, disorders of neurotransmitters, and release of inflammatory mediators (Kim-Han et al., 2006). A number of factors are triggered after ICH, resulting in hematoma and deep brain cell death. The loss of nerve cells is a major cause of mortality in ICH patients. Programmed cell death plays an important role in cerebral hemorrhage (Bobinger et al., 2018). Ferroptosis, a programmed cell death-like process, has been identified after ICH, but the mechanism of ferroptosis in cerebral hemorrhage remains unclear.

Ferroptosis was first described by Stockwell in 2012 as a type of cell death that differs from apoptosis, and its distinguishing feature is iron-dependent accretion of lipid peroxidation by reactive oxygen species (ROS) (Dixon et al., 2012). Recent studies have revealed that this process is related to the cell death mechanisms involved in ICH (Bao et al., 2020). It has been found that the expression levels of some key molecules change after ferroptosis. In particular, some studies have revealed that the level of phospho*-*mitogen-activated protein kinase 1 (p-MAPK1) significantly increases after ICH, and the MAPK/ERK pathway is involved in ferroptosis regulation of ICH (Chen S. et al., 2019; Su et al., 2019). With respect to the downstream mediators of ferroptosis, a previous study found that cyclooxygenase 2 (COX-2), which is encoded by the *PTGS-2* gene, significantly increased in mice after ICH, and it was inhibited by fe-1 (Chen B. et al., 2019). Activating transcription factor 4 (ATF4) is activated in the ferroptosis-induced transcriptional response in neurons, and the neurons of the ATF4 deletion germline are resistant to homocysteine-induced ferroptosis (Karuppagounder et al., 2016; Chen et al., 2017). However, many ferroptosis genes have not yet been found, so further studies of ferroptosis-related genes are needed.

At present, there have been no bioinformatics-based studies on the mechanism of ferroptosis genes after ICH. Therefore, we used data mining and data analysis techniques to screen differentially expressed genes (DEGs) in perihematomal tissue (PH) and contralateral normal tissue of ICH patients. These DEGs were then intersected with the ferroptosis dataset to obtain ferroptosis DEGs. Moreover, to identify crucial biomarkers and establish the pathogenesis of ICH at the molecular level, we investigated key miRNAs and lncRNAs that may play principal roles in ICH. We also constructed a rat ICH model and an intervention model using treadmill training to test the above-mentioned hypothesis. Our results will help to understand ferroptosis after ICH and provide new thoughts for clinical diagnosis and treatment of ICH.

## Materials and Methods

### Animals

Adult male Sprague–Dawley rats (2 months old) were purchased from Qingdao Peng Yue Animal Husbandry Co., Ltd., and kept under a 12-h light/dark cycle in the same colony room, with controlled temperature and humidity. The animals were randomly assigned into the following three groups: (1) sham (*n* = 7): rats that underwent surgery without ICH; (2) ICH (*n* = 7): the ICH group, animals that received autologous blood injection; and (3) ICH+ early exercise (*n* = 7): rats that received additional treadmill training after ICH. The animal study was reviewed and approved by the Ethics Committee of the Affiliated Hospital of Qingdao.

### Intracerebral Hemorrhage Model

As in previous studies, cerebral hemorrhage was induced in the right caudate nucleus (Wu et al., 2016). The rats were anesthetized with isoflurane and fixed in a stereotaxic apparatus. A hole was perforated 4.0 mm laterally to the median line of the brain and 0.1 mm anterior to the bregma, and 1.5 μl saline mixed with 0.38U of bacterial collagenase (type IV; Sigma-Aldrich, St. Louis, MO, United States) was injected via a 5.0-mm needle below the plane of the cranial bones.

### Treadmill Training

The exercise program used throughout the study was slightly adjusted compared with the method used in previous studies (Tamakoshi et al., 2018). In short, 3 days before the operation, the rats were acclimated to the treadmill training environment for 20 min at 1–2 m/min. We ensured that the rats were able to carry out normal exercise training in the next exercise protocol and excluded the rats that did not participate in the training. On postoperative day 2, the ICH group did not carry out treadmill training and lived in the above-mentioned conventional feeding environment for the next 2 weeks. In contrast, the ICH+ early exercise group was subjected to the training for 14 days. The training time was set to 30 min/day, with the training speed set at 6 M/min on the first day and 10 M/min thereafter.

### Microarray Data

The clinical information of patients with cerebral hemorrhage was obtained from Gene Expression Omnibus (GEO)^1^. We downloaded the dataset GSE24265 stored by Rosell from the GEO (Rosell et al., 2011). This dataset includes microarray data of perihematoma tissues and contralateral corresponding parts (white matter and gray matter) of four patients with ICH (Liu et al., 2019). This dataset was used for further analysis and mining. In this experiment, the microarray data of 11 samples, including perihematomal tissue (PH) and the corresponding contralateral white and gray matter, were obtained from public databases, so the consent of patients and ethics committee approval were unnecessary.

### Differential Expression Analysis

GEO2R, an online analysis tool, was used to perform the analysis of differential expression (Barrett et al., 2013). The expression profiles of PH and normal tissue were compared to identify the DEGs. *T* test was employed to determine *p*-values and adjusted *p*-values in the differential gene expression (DGE) analysis.

Genes retained from diverse tissues were selected using the following criteria: a | log2 (fold-change)| > 1 and adjusted *P*-value < 0.05. We also obtained a dataset that included 265 genes from the Ferroptosis Database (FerrDb; zhounan.org) and intersected it with GSE24265 to identify ferroptosis DEGs. The online tool Venny2.1 was employed to generate a Venn diagram of DEGs, and a heat map of DEGs was drawn using Heml Software.

### Functional Enrichment Analysis

Functional enrichment analysis of DEGs was performed using DAVID 6.8, Metascape, and WebGestalt. These different enrichment analysis tools have different algorithms, which can play the role of mutual verification. Gene set enrichment analysis (GSEA) is a method to sort genes according to the degree of differential expression of two samples (Subramanian et al., 2005). It is a method to analyze the whole genome expression profile chip data and compare genes with predefined gene sets (Debrabant, 2017). We uploaded the ferroptosis DEGs to the GSEA of WebGestalt for further study. The GSEA of WebGestalt first filtered the gene sets according to the number of genes contained, with a minimum number of seven genes and a maximum number of 2,000 genes per gene set by default. Kyoto Encyclopedia of Genes and Genomes (KEGG) analysis was obtained from the GSEA of WebGestalt. A common enrichment tool, David v6.8, was also used for enrichment analysis, focusing on the KEGG pathway analysis and KEGG enrichment analysis to find the pathways where the target gene is involved and analyze the significance of each pathway. We uploaded the obtained ferroptosis DEGs in Metascape, an online tool for gene function annotation analysis. Annotation of biological processes was performed by Metascape, using the genes that were shared between GSE24265 and the ferroptosis dataset. Moreover, the biological pathways of miRNAs were analyzed in an enrichment analysis tool, Funrich. The difference was statistically significant at *P* < 0.05.

### Protein–Protein Interaction Network Analysis

To predict protein–protein interactions (PPIs), STRING, an online database which can retrieve the interaction between a group of proteins, was utilized in the PPI network analysis (Szklarczyk et al., 2017). Cytoscape network visualization was obtained with interaction scores > 0.4. The nodes represented genes, and the edges represented the links between the genes. In addition, the PPI network was built and visualized by Cytoscape v3.6.0 software.

Molecular complex detection (MCODE) was used for clustering analysis of gene networks to identify key PPI network modules (Bader and Hogue, 2003). The function of MCODE is to select the key sub-networks, i.e., modules. A PPI module refers to a PPI module where one module points to one function. In a module, different genes have different module scores, and key genes can be selected according to the scores. In order to identify the key modules, *P* < 0.05 was considered to show a significant difference.

### Gene–miRNA Interaction Networks

In addition to the abovementioned analytical tools, we also used miRWalk 2.0 to predict targeted pivotal miRNAs and build the gene–miRNA interaction networks that are related to cluster 1 (Dweep et al., 2011). We intersected the predicted results of the MiRTarBase and miRWalk database to ensure the accuracy of the results. We screened miRNAs that target more than two genes.

### miRNA–lncRNA Prediction

lncRNAs, which are upstream molecules of miRNAs, were identified using StarBase v2.0 (Dweep et al., 2011). We selected the cross-linked diagram showing the interaction of the calculated results of every miRNA to confirm relevant lncRNAs.

### qRT-PCR Analysis

On postoperative day 15 (sham: *n* = 7; ICH: *n* = 7; ICH + early exercise: *n* = 7), PH was extracted, and qRT-PCR was performed. Briefly, total RNA was collected from the PH of rats using TRIzol. RNA purity was tested using Quantus Fluorometer. First, total RNA was used for reverse transcription reaction. Then, the pre-amplified cDNA samples were mixed with One-Step SYBR PrimeScript PLUS RTPCR Kit. Finally, the reaction was conducted on AriaMx HRM. GAPDH was used as a positive control, and the comparative Ct method was used to calculate each sample (Table 1).

**TABLE 1:** Specific primers used for quantitative real-time PCR.

**Gene**	**Forward (5′–3′)**	**Reverse (5′–3′)**
MAPK1	CCTTGACCAGCTGAATCACATC	TCAGCGTTTGGGAACAACCT
MAPK8	GCTGGTGATAGATGCGTCCAA	TCCTCTATTGTGTGCTCCCTTTC
SLC2A1	CAGCTGCCCTGGATGTCCTA	GAAGCCAGCCACAGCAACA
TNFAIP3	CATCCTCAGAAGACCCATCATTG	CCAAGGACGATGGGATATCTGT
ATF4	AGGTGGCCAAGCACTTCAAA	GGTCCATTTTCTCCAACATCCA
GAPDH	CAGCCGCATCTTCTTGTGC	GGTAACCAGGCGTCCGATA

### Statistical Analysis

GraphPad Prism 7.0 software was used to draw graphics and perform statistical analysis. All of the data are shown as mean ± standard deviation (SD). One-way analysis of variance (ANOVA) was used for statistical analysis of sham, ICH, and ICH + early exercise groups, followed by Tukey’s multiple-comparisons *post hoc* tests. A *t*-test was employed to determine *p*-values and adjusted *p*-values in the DGE analysis, where *p*-values were adjusted by FDR. The difference was statistically significant at the level of *P* < 0.05.

## Results

### Ferroptosis DEGs

**FIGURE 1:**
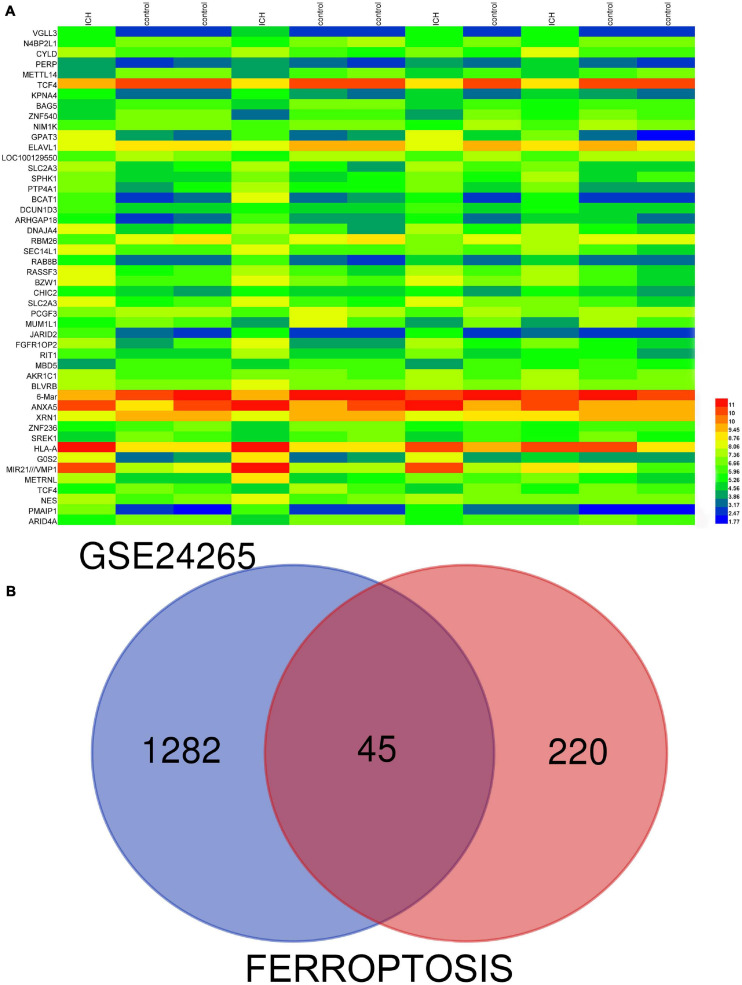
**(A)** There were 1,327 differentially expressed genes (DEGs) in perihematomal tissue and normal tissues. The first 50 differentially expressed genes were shown in the heat map, with red representing significantly up-regulated genes and blue representing significantly down-regulated genes in the samples. **(B)** Venn diagram of ferroptosis differentially expressed genes. We intersected ferroptosis dataset with GSE24265 to identify ferroptosis DEGs.

GSE24265, the microarray expression profiling dataset, was downloaded from the GEO database, and DEGs were obtained by comparing PH and the corresponding gray and contralateral white matter. We also gained the dataset including 265 genes from the Ferroptosis Database (FerrDb) and intersected them with GSE24265 to identify ferroptosis DEGs. We found altogether 40 upregulated and five downregulated genes (Table 2). The heat map and Venn diagram of the DEGs are shown in Figure 1. The DEGS were further classified as ferroptosis driver, ferroptosis suppressor, and ferroptosis marker via the FerrDb online tool (Table 3).

**TABLE 2:** Ferroptosis differentially expressed genes of intracerebral hemorrhage.

**Gene symbol**	***P*-value**	**Fold change**	**Gene title**	**ID**
**Upregulated genes**				
CD44	0.0121	1.87	CD44 molecule (Indian blood group)	204490_s_at
HSPB1	0.00249	1.52	Heat shock protein family B (small) member 1	201841_s_at
BID	0.0351	1.01	BH3 interacting domain death agonist	211725_s_at
PANX1	0.0292	1.04	Pannexin 1	204715_at
ACSL3	0.00585	1.19	Acyl-CoA synthetase long-chain family member 3	201662_s_at
SQSTM1	0.0343	1.9	Sequestosome 1	244804_at
ATF3	0.00167	1.07	Activating transcription factor 3	1554980_a_at
PLIN2	0.00476	3.12	Perilipin 2	209122_at
CRYAB	0.0327	1.42	Crystallin alpha B	209283_at
SLC2A3	2E-05	2.35	Solute carrier family 2 member 3	216236_s_at
NCF2	0.00424	2.54	Neutrophil cytosolic factor 2	209949_at
NRAS	0.0211	1.36	Neuroblastoma RAS viral oncogene homolog	202647_s_at
FTL	0.00021	1.38	Ferritin light chain	213187_x_at
SCD	0.0245	1.14	Stearoyl-CoA desaturase	211162_x_at
STAT3	0.00035	1.37	Signal transducer and activator of transcription 3	208992_s_at
CAPG	0.0134	1.23	Capping actin protein, gelsolin-like	201850_at
PTGS2	0.0326	1.9	Prostaglandin-endoperoxide synthase 2	1554997_a_at
DUSP1	0.0222	1.4	Dual specificity phosphatase 1	201041_s_at
GCH1	0.00934	1.98	GTP cyclohydrolase 1	204224_s_at
GABPB1	0.0046	1.43	GA binding protein transcription factor beta subunit 1	206173_x_at
VEGFA	0.004	1.53	Vascular endothelial growth factor A	210513_s_at
ATF4	0.00232	1.14	Activating transcription factor 4	200779_at
TNFAIP3	0.00036	3.04	TNF alpha induced protein 3	202644_s_at
IL6	0.00848	2.58	Interleukin 6	205207_at
SLC2A1	0.00126	1.11	Solute carrier family 2 member 1	201250_s_at
HMOX1	0.00117	2.21	Heme oxygenase 1	203665_at
DDIT4	0.0336	1.29	DNA damage inducible transcript 4	202887_s_at
MAPK1	0.00438	1.38	Mitogen-activated protein kinase 1	1552263_at
CXCL2	0.00445	3.99	C-X-C motif chemokine ligand 2	209774_x_at
DDIT3	0.00075	1.45	DNA damage inducible transcript 3	209383_at
HSPA5	0.0063	1.7	Heat shock protein family A (Hsp70) member 5	211936_at
CAV1	0.00487	1.47	Caveolin 1	203065_s_at
CEBPG	0.00961	1.15	CCAAT/enhancer binding protein gamma	204203_at
AKR1C1	0.0001	1.07	Aldo-keto reductase family 1 member C1	216594_x_at
RPL8	0.00027	1.28	Ribosomal protein L8	200936_at
SESN2	0.0388	1.04	Sestrin 2	223195_s_at
SAT1	0.00078	2.18	Spermidine/spermine N1-acetyltransferase 1	213988_s_at
**Downregulated genes**				
STMN1	0.00754	−1.43	Stathmin 1	1552803_a_at
PRKAA2	0.0093	−1.31	Protein kinase AMP-activated catalytic subunit alpha 2	227892_at
SLC2A12	0.0111	−1.48	Solute carrier family 2 member 12	244353_s_at
MAPK8	0.046	−1.07	Mitogen-activated protein kinase 8	229664_at
KLHL24	0.0104	−1.41	Kelch-like family member 24	226158_at

**TABLE 3:** The ferroptosis differentially expressed genes were divided into ferroptosis driver, suppressor, and marker.

**Suppressor**	**Driver**	**Marker**
AKR1C2, GCH1, AKR1C1, PLIN2, SCD, CD44, CAV1, STAT3, ACSL3, HMOX1, ATF4, SESN2, SQSTM1, HSPA5, HSPB1	TNFAIP3, HMOX1, ATF4, MAPK1, MAPK8, PRKAA2, ATF3, BID, NRAS	NCF2, PTGS2, IL6, CXCL2, SLC2A1, DUSP1, VEGFA, CEBPG, KLHL24, DDIT3, DDIT4, GABPB1, SLC2A12, STMN1, SLC2A14, SLC2A3

### Enrichment Pathway and Analysis of Ferroptosis DEGs

**FIGURE 2:**
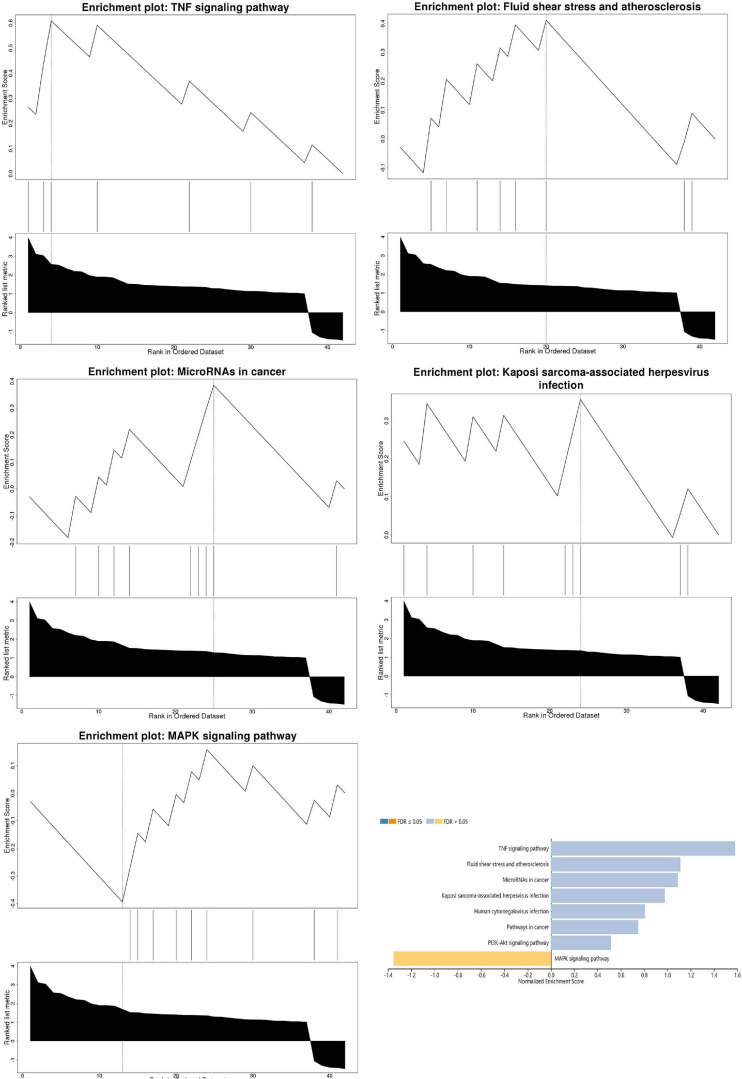
The result of the enrichment gene dataset analysis indicated that the genes significantly enriched were involved in the TNF signaling pathway, MAPK signaling pathway, fluid shear stress, atherosclerosis, and Kaposi’s sarcoma-associated herpesvirus infection. Gene set enrichment analysis of WebGestalt first filtered the gene set according to the number of genes contained in the gene set, with a minimum number of seven genes and a maximum number of 2,000 genes by default.

**FIGURE 3:**
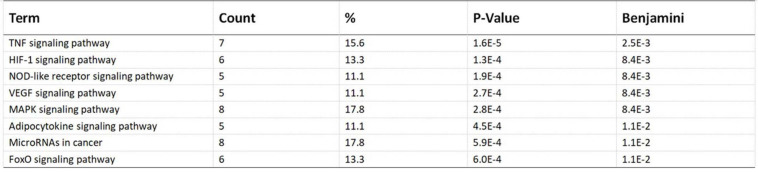
Top eight biological pathways were selected and shown according to enrichment score. The TNF signaling pathway was significantly enriched.

**FIGURE 4:**
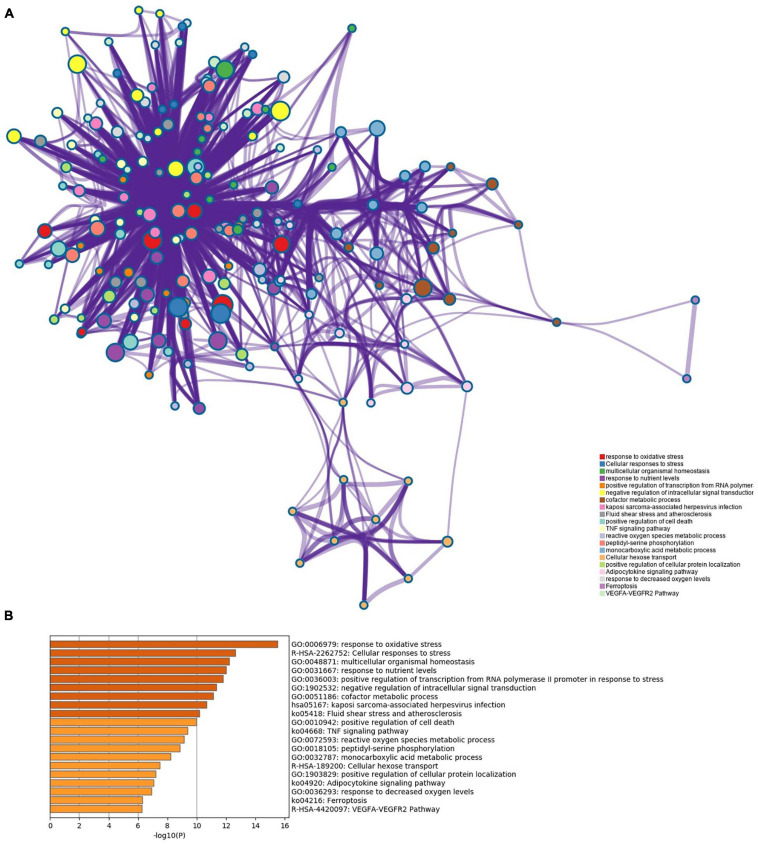
**(A)** Network of enriched terms. **(B)** The Metascape drew a bar chart of 20 biological pathways based on the *P*-value and the percentage of genes, among which biological pathways with *P*-value < 0.01 are statistically significant. The results showed that the biological processes that were significantly enriched were in response to oxidative stress.

Pathway enrichment analyses of DEGs were performed using the online tools Metascape, WebGestalt, and DAVID. First, we uploaded the related information of DEGs in the PH samples and normal samples to the WebGestalt software. The result of the enrichment gene dataset analysis indicated that the genes significantly enriched were involved in the TNF signaling pathway, MAPK signaling pathway, fluid shear stress, atherosclerosis, and Kaposi’s sarcoma-associated herpesvirus infection (Figure 2). DAVID was utilized to analyze the biological pathways and processes of 45 DEGs in ICH samples. KEGG functional analysis revealed that the TNF signaling pathway, HIF-1 signaling pathway, MAPK signaling pathway, and NOD-like receptor signaling pathway were significantly activated in the gene sets (Figure 3). Second, 45 DEGs were then uploaded to Metascape, and it was shown that the biological processes were remarkably enriched in response to oxidative stress, multicellular organismal homeostasis RNA polymerase II promoter in response to stress, and cellular response to stress. The biological process was significantly activated in response to stress. The biological pathway was significantly activated in the TNF signaling pathway, fluid shear stress, atherosclerosis, and Kaposi’s sarcoma-associated herpesvirus infection (Figure 4). Notably, the TNF signaling pathway was the major biological pathway involved and was identified by Metascape, WebGestalt, and DAVID.

### Protein–Protein Interaction Network Analysis of Ferroptosis DEGs

**FIGURE 5:**
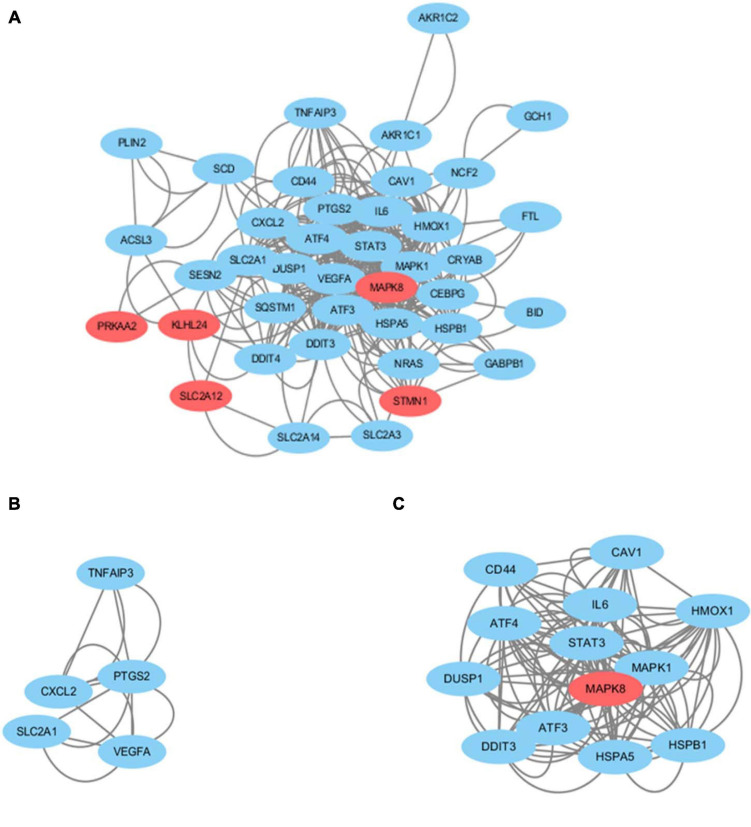
**(A)** Cytoscape network visualization of the 40 nodes and 330 edges that were obtained with interaction scores > 0.4 according to the STRING online database. The nodes represent genes and the edges represent links between genes. Red represents downregulated genes, and blue represents upregulated genes. Two key modules were identified by MCODE, which was used to identify network gene clustering. **(B)** Cluster 2. **(C)** Cluster 1.

**FIGURE 6:**
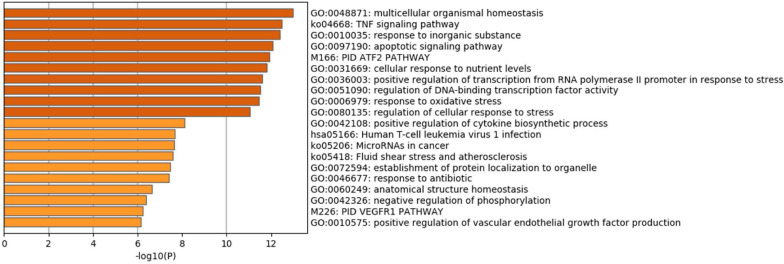
Functional enrichment analysis of cluster 1. The TNF signaling pathway was significantly enriched.

We obtained the PPI network containing 40 nodes and 330 edges. Five of the 45 genes were not related to other molecules and did not form a molecular network. The network was set to the default cutoff (interaction score > 0.4) in the STRING online database. Genes were represented by nodes, and the interactions between the genes were indicated by edges. The upregulated genes were marked in blue, while the downregulated genes were labeled in red. MCODE, the application from Cytoscape, was used for clustering analysis of gene networks to draw the key modules (Table 4). Two key modules with one downregulated gene (*MAPK8*) and 17 upregulated genes were established (Figure 5). These 18 genes were the key genes screened by MCODE. Genes of interest were selected from the genes identified by key module analyses involved in the TNF pathways with the highest MCODE scores, including *MAPK1*, *MAPK8*, *TNFAIP3*, *ATF4*, and *SLC2A1*. Furthermore, the functional analysis for cluster 1 using Metascape showed that these 18 genes were mainly involved in multicellular organismal homeostasis, TNF signaling pathway, and inorganic substance (Figure 6).

**TABLE 4:** Molecular complex detection was used to process the data downloaded from the STRING to further mining gene clusters.

**Cluster**	Score (density * # of nodes)	**Nodes**	**Edges**	**Node IDs**
1	10	13	120	CD44, CAV1, ATF4, IL6, HMOX1, DUSP1, STAT3, MAPK1, MAPK8, DDIT3, ATF3, HSPA5, HSPB1
2	3.5	5	14	TNFAIP3, PTGS2, CXCL2, SLC2A1, VEGFA

### Further MiRNA Interaction and Mining

**FIGURE 7:**
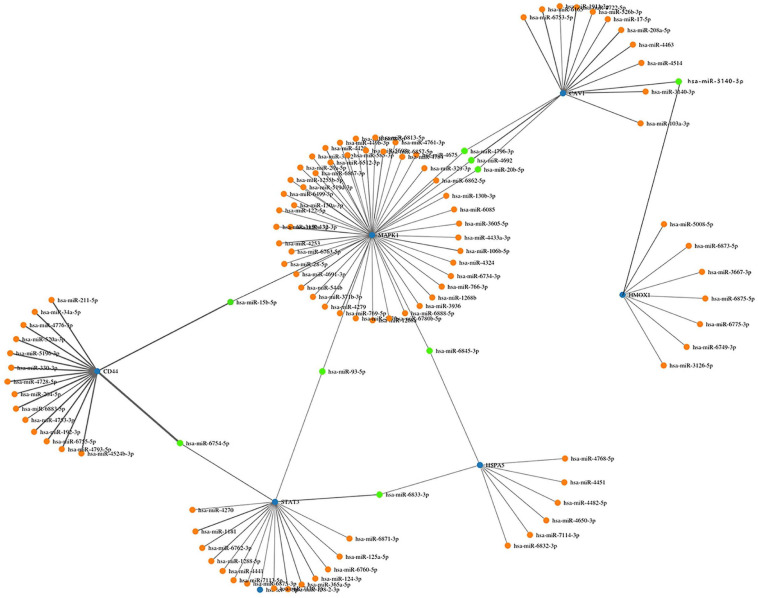
Interaction network between genes of cluster 1 and its targeted miRNAs. Genes were colored in blue, miRNAs were colored in orange, and the cross-linked genes were colored in green.

**FIGURE 8:**
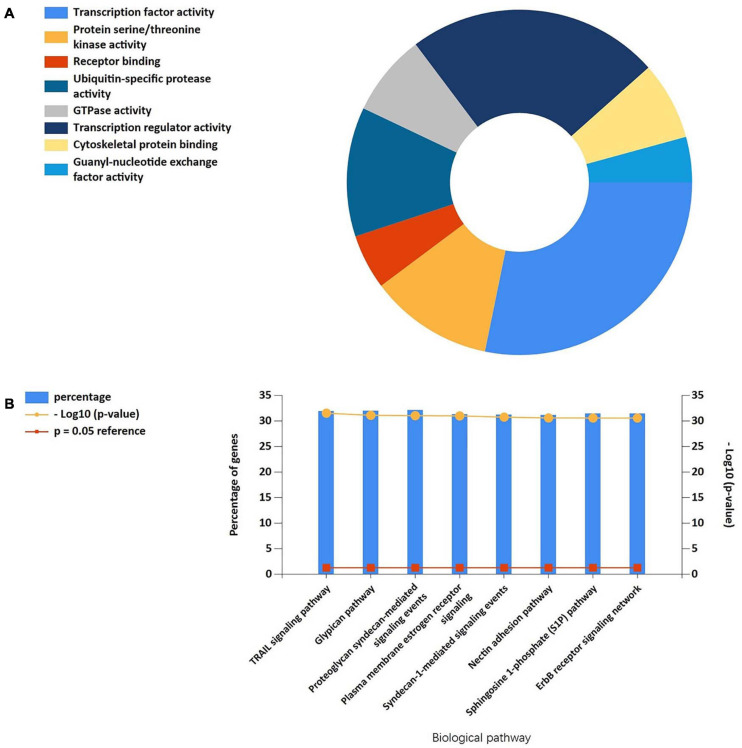
**(A)** The molecular function of cluster 1-related miRNAs was significantly enriched in the transcription factor activity, protein serine kinase activity, and receptor binding. **(B)** Biological pathways were enriched in the TRAIL signaling pathway, Glypican pathway, and proteoglycan syndecan-mediated signaling events.

We screened 13 genes in cluster 1 and performed gene–miRNA analysis by using miRWalk 2.0 software. The cross-linked miRNAs were selected by miRWalk and miRTarBase databases to ensure the accuracy and reliability of our results. The following criteria were used in filtering the results: *P* < 0.05, seed sequence lengths > 7, and 3′UTR as the target gene-binding regions (Figure 7). The miRNAs with higher amounts of cross-linked genes (≥2) are shown in Table 5. A total of 300 miRNA expression genes were uploaded to Funrich, and the results of the enrichment analysis indicated that the molecular function was significantly enriched in transcription factor activity, protein serine kinase activity, and receptor binding. The biological pathways enriched included the TRAIL signaling pathway, glypican pathway, and proteoglycan syndecan-mediated signaling events (Figure 8).

**TABLE 5:** miRNAs and its target genes.

**miRNA**	**Genes targeted by miRNA**	**Gene count**
miR-15b-5p	CD44, MAPK1	2
miR-20b-5p	MAPK1, CAV1	2
miR-93-5p	STAT3, MAPK1	2
miR-6754-5p	CD44, STAT3	2
miR-6833-3p	HSPA5, STAT3	2
miR-6845-3p	MAPK1, HSPA5	2
miR-4692	CAV1, MAPK1	2
miR-4796-3p	MAPK1, CAV1	2
miR-5193	HMOX1, CAV1	2

### lncRNA Prediction

Nine miRNAs had higher amounts of cross-linked genes (≥2). Only *hsamiR-15b-5P*, *hsa-miR-93-5P*, and *miR-20b-5p* had the upstream molecule lncRNA and had higher reliability, all targeting *MAPK1*. The corresponding lncRNAs of *hsa-miR-15b-5P*, *hsa-miR-93-5P*, and *miR-20b-5p* were predicted with StarBase 2.0. The highest reliability (very high stringency, >5) was selected as the criterion. After cross-linking, nine lncRNAs targeting three key miRNAs were revealed, namely, *SNHG16*, *XIST*, *AC084219.4*, *RP11-379K17.11*, *CTC-444N24.11*, *GS1-358P8.4*, *CTB-89H12.4*, *RP4-773N10.5*, and *FGD5-AS1.*

### Potential Biomarker Expression by qRT-PCR

**FIGURE 9:**
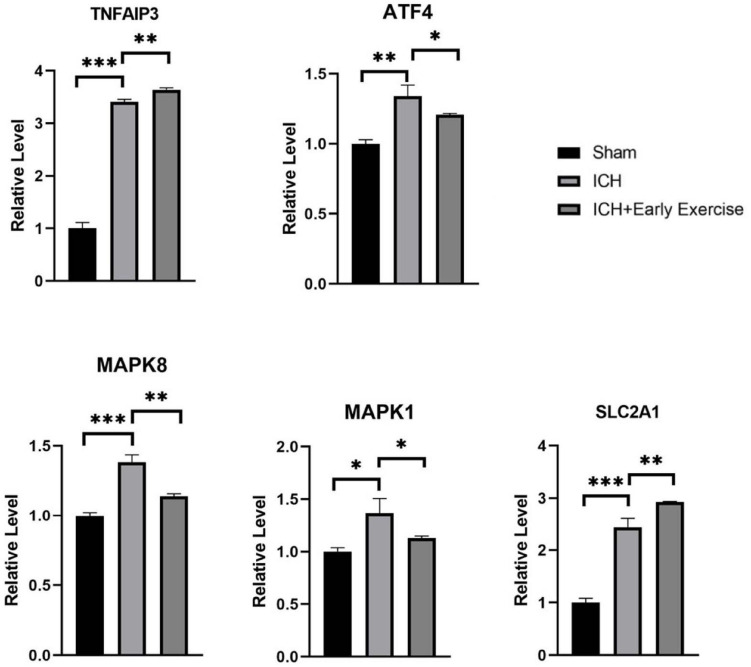
qRT-PCR results show that the expression levels of MAPK1 (*P*-value = 0.011), SLC2A1 (*P*-value = 0.0002), TNFAIP3 (*P*-value < 0.0001), and ATF4 (*P*-value = 0.0022) were obviously higher, and MAPK8 (*P*-value = 0.0003) was lower in intracerebral hemorrhage rats than that of healthy controls. After treadmill training, SLC2A1 (*P*-value = 0.0087) and TNFAIP3 (*P*-value < 0.0028) were obviously upregulated, and ATF4 (*P*-value = 0.0468), MAPK8 (*P*-value = 0.0017), and MAPK1 (*P*-value = 0.0408) were downregulated by comparing the treatment group and the model groups.

Five genes were verified. They had high reliability and were mostly related to TNF signaling and response to stress. *SLC2A1*, *TNFAIP3*, *ATF4*, *MAPK1*, and *MAPK8* were identified by MCODE as the genes with high MCODE score in the two clusters. Then, the filtered biomarkers, including *MAPK1*, *MAPK8*, *TNFAIP3*, *ATF4*, and *SLC2A1*, were verified in ICH samples using qRT-PCR. The results indicated that the expression levels of *MAPK1*, *ATF4*, *SLC2A1*, and *TNFAIP3* were visibly higher and that the expression of *MAPK8* was lower in ICH rats than in the sham controls. After the exercise training, *MAPK1*, *MAPK8*, and *ATF4* showed low expression levels compared with the ICH group. In contrast, *TNFAIP3* and *SLC2A1* were upregulated by exercise training (Figure 9).

## Discussion

The present study identified the key genes involved in ferroptosis and further explored the mechanisms of ferroptosis in ICH. Our study obtained 45 DEGs, including six downregulated genes and 39 upregulated genes, from the intersection of DEGs of the datasets GSE24265 and FerrDb. Then, pathway enrichment analyses of DEGs were performed using the online tools Metascape, GSEA, and DAVID, and the results indicated that these genes are mainly involved in stress responses and the TNF signaling pathway. The changes in ferroptosis gene expression after ICH followed by treadmill training were identified, suggesting that exercise may influence ferroptosis in the treatment of ICH and proving the reliability of the bioinformatics analysis. Moreover, the regulatory network of *MAPK1* molecules can affect the TNF signaling pathway after ICH. The study also identified several genes that had not yet been mentioned in the area of ICH and ferroptosis. This study may provide effective reference for the pathological mechanism of ICH from the perspective of bioinformatics analysis.

Ferroptosis is characterized by the intracellular collection of lipid ROS, which are closely related and eventually result in the oxidation of lipids, thereby leading to cell membrane injury and cell death. Ferroptosis is associated with oxidative stress produced by the undue collection of ROS during aerobic metabolism (Dixon et al., 2012). After oxidative stress, some signaling pathways are activated, such as the MAPK pathway. According to previous studies, the MAPK pathway and the NF-κB pathway may induce the production of free radicals after ICH, which can induce ferroptosis and apoptosis; in that context, antioxidant therapy can decrease the ferroptosis of neurons as well as apoptosis after ICH (Haddad and Land, 2002; Speer et al., 2013). A previous study confirmed that the MAPK pathway is activated following iron accumulation, and the suppression of MAPK activation improves functional results and reduces neuronal cell death (Yu and Richardson, 2011). Therefore, together with the induction of ferroptosis, the MAPK signaling pathway and the NF-κB pathway might be activated to promote the generation of ROS, aggravating cell injury and resulting in a vicious circle.

The TNF signaling pathway plays a significant role in inflammation and oxidative stress (Fischer and Maier, 2015). In the binding of TNF to TNFR1, the signaling complex I adjusts the NF-κB pathway via the activation of the IKK complex, causing the translocation of NF-κB to the nucleus, which changes the expression of cell adhesion molecules, proinflammatory cytokines, and ROS-generating enzymes (Walczak, 2011). In contrast, TNFR2 does not possess a death domain and thus indirectly leads to cell death. Signaling pathways activated by TNFR2 include the NF-κB and JNK pathways, canonical and non-canonical (Cabal-Hierro et al., 2014). TNFα/TNFR2 signaling can regulate the enzyme TNFAIP3/A20 to reduce IL-17A expression in conventional T cells, thereby exhibiting an anti-inflammatory effect (Urbano et al., 2019). Therefore, the TNF signaling pathway is also a double-edged sword and has potential research value. A previous experiment in mice showed that TNF-α treatment notably raised the level of glutamate, resulting in stress in astrocytes during neuroinflammation (Wang et al., 2017). Neuroinflammation through glutamate toxicity is considered an important target in the pathophysiology of ICH-induced nervous system injury (Shah et al., 2016). Glutamate toxicity closely involves ferroptosis. Therefore, the TNF signaling pathway may play an important role in ferroptosis following hemorrhage stroke.

In order to test and verify our hypothesis, enrichment analyses containing modular enrichment analysis and gene sets were performed by different bioinformatics analysis tools. DAVID and GSEA of WebGestalt revealed that the TNF signaling pathway was significantly enriched by KEGG pathway analysis, and there were seven genes correlating with this pathway, namely, *IL6*, *MAPK8*, *TNFAIP3*, *MAPK1*, *PTGS2*, *CXCL2*, and *ATF4*. The Metascape analysis also revealed that oxidative stress was markedly activated in the Gene Ontology process, and there were 15 genes associated with this pathway, namely, *ATF4*, *CRYAB*, *DUSP1*, *GCH1*, *HBA1*, *HMOX1*, *HSPB1*, *IL6*, *NCF2*, *PRKAA2*, *MAPK1*, *MAPK8*, *PTGS2*, *TNFAIP3*, and *SESN2.*

The genes of interest were selected from the genes identified by the key module analyses involved in the TNF pathway with the highest MCODE scores, including *MAPK1*, *MAPK8*, *TNFAIP3*, *ATF4*, and *SLC2A1. MAPK1* is a serine/threonine kinase that plays important roles in several cellular processes. It has been shown that P-MAPK1 expression considerably increased and promoted neuroinflammation after ICH (Haddad and Land, 2002). The suppression of ferroptosis ameliorated hemoglobin-induced cell death by inhibiting MAPK/ERK1 (Codenotti et al., 2018). MAPK8, which is also known as C-JUN N-terminal kinase (JNK), is a member of the MAPK signaling pathway. A previous study on ICH suggested that the JNK pathway could be initiated, with upregulation of pJNK (Hibi et al., 1993; Pei et al., 2016). Other studies indicated that JNK is involved in ferroptotic cell death in mouse cortex and PC-12 cells (Qin et al., 2020). TNFAIP3, an important anti-inflammatory factor, can repress the expression of inflammatory NF-κB signaling and promote cell survival (Catrysse et al., 2014). A previous study suggested that TNFAIP3 could prevent ICH-caused damaging inflammation of the brain. However, TNFAIP3 overexpression increased oxygen free radical generation and enhanced ferroptosis induced by erastin (Meng et al., 2017; Xiao et al., 2019). In the nervous system, ATF4 is a key redox-regulated, pro-death transcriptional activator that propagates death responses to oxidative stress in stroke (Ameri and Harris, 2008). It has been shown that ATF4 increases the expression of transcriptional activator CHOP (DDIT3) and caspase-12, which promote apoptosis and reduce neuronal survival after brain hemorrhage (Wu et al., 2020). Upregulated ATF4 increases the expression of HSPA5 and activity of glutathione peroxidase 4 (GPX4), thus protecting glioma cells from ferroptosis (Chen Y. et al., 2019). Insufficiency of the SLC2A1 gene and the weakness of its translated product, glucose transporter-1 (Glut1) protein, affect glucose uptake, disrupt brain function, and cause neurodevelopmental disorders (Veys et al., 2020). A previous study demonstrated that lymphoid-specific helicase inhibited ferroptosis, partly through Glut1 (Jiang et al., 2017). These data suggest that the changes in *MAPK1*, *MAPK8*, *TNFAIP3*, *ATF4*, and *SLC2A1* gene expression may be the basis for the increased incidence of ferroptosis after ICH.

miRNAs are endogenous non-coding RNA molecules that target the 3′UTR region of a gene and can regulate gene expression to degrade or inhibit the translation of the target gene (Sun et al., 2015). In our study, we identified nine miRNAs targeting at least two genes. However, among these nine miRNAs, only *hsa-miR-15b-5p*, *hsa-miR-93-5p*, and *miR-20b-5p* can forecast the relevant upstream lncRNAs. In previous studies, *miR-20b-5p* and *miR-15b-5p* in ICH were limited, while *miR-15b-5p* was upregulated in meningitis (de Queiroz et al., 2018). *miR-93-5p* is significantly downregulated in ICH (Cheng et al., 2020). Our results indicated that the mRNA–miRNA–lncRNA connection chain may exist in patients with ICH. Further experiments should be carried out to determine the specific molecular mechanism.

lncRNAs has also been affirmed as a new target for disease therapy and diagnosis (Zhang and Wang, 2019). The expression level of miRNA is also affected by its upstream molecules, lncRNAs (Militello et al., 2017). While *H19*, *Igsf7*, and *Lilrb4* are upregulated, *Irs4* and *LOC103691048* are downregulated in ICH patients (Kim et al., 2019). Some studies indicated that dysregulated lncRNAs were involved in immune response, immune system process, the type I interferon signaling pathway, and defense response (Suarez et al., 2020). This study showed that *hsa-miR-15b-5p*, *hsa-mir-93-5p*, and *miR-20b-5p* were highly correlated with *MAPK1*; thus, *SNHG16*, *XIST*, *AC084219.4*, *RP11-379K17.11*, *CTC-444N24.11GS1-358P8.4*, *CTB-89H12.4*, *RP4-773N10.5*, and *FGD5-AS1* may be potentially used as novel biomarkers.

In order to further verify our conclusions, we also performed *in vivo* experiments, generated the ICH model, and conducted exercise training for the treatment of cerebral hemorrhage. PCR analysis revealed that *MAPK8* was downregulated in ICH, whereas *ATF4*, *MAPK1*, *SLC2A1*, and *TNFAIP3* were upregulated, and these results are concordant with the findings of previous research. It has been revealed that early exercise training can ameliorate neuron cell injury after ICH (Macko et al., 2005). Our study revealed that exercise training may inhibit the MAPK signaling pathway and reduce the expression of *MAPK1* and *MAPK8. ATF4* was also inhibited as a ferroptosis driver gene. In contrast, the levels of *TNFAIP3* and *SLC2A1* increased after exercise training. It may be that *TNFAIP3* is an antineuritic factor in ICH, and *SLC2A1* is a beneficial energy transporter. Therefore, exercise training as an antioxidant therapy may ameliorate nerve injury after cerebral hemorrhage via the above-mentioned ferroptosis genes. Furthermore, the PCR results of our *in vivo* experiments preliminarily verified the rationality of our bioinformatics results.

The current study identified several genes that are closely associated with ferroptosis after cerebral hemorrhage, some of which had not yet been mentioned in the context of cerebral hemorrhage. These novel genes provide new concepts in ferroptosis after ICH. We found that the stress response pathway, especially the TNF signaling pathway, is extremely important in ferroptosis after cerebral hemorrhage. In addition, molecules significantly associated with TNF signaling, *MAPK1* and its associated miRNA and lncRNAs, were found to be potential biomolecules in the ferroptosis pathway after cerebral hemorrhage. We also treated ICH rats by treadmill training to verify the results of our analysis and compared these with healthy controls. We showed that MAPK8 was downregulated, whereas *ATF4*, *MAPK1*, *SLC2A1*, and *TNFAIP3* were upregulated in ICH rats. After the exercise intervention, the anti-inflammatory molecule *TNFAIP3* and energy regulation factor *SLC2A1* were upregulated, while *MAPK8*, *MAPK1*, and *ATF4* were downregulated, which suggests that treadmill training may influence ferroptosis. The identification of these genes can provide new therapeutic targets for ferroptosis after cerebral hemorrhage, and the regulatory network of *MAPK1* molecules can affect the TNF signaling pathway after cerebral hemorrhage.

## Data Availability Statement

Publicly available datasets were analyzed in this study. This data can be found here: FerrDb (http://www.zhounan.org/ferrdb/) and https://www.ncbi.nlm.nih.gov/geo/query/acc.cgi?acc=GSE24265.

## Ethics Statement

The animal study was reviewed and approved by The Ethics Committee of Affiliated Hospital of Qingdao University.

## Author Contributions

QW and TL designed the experiments. YW, TL, XL, and YC performed the experiments. TL, XL, PM, and GZ wrote the manuscript and analyzed the data. All authors contributed to the article and approved the submitted version.

## Conflict of Interest

The authors declare that the research was conducted in the absence of any commercial or financial relationships that could be construed as a potential conflict of interest.

## Footnotes

1 https://www.ncbi.nlm.nih.gov/geo/
