# Supplementary material for: Bioinformatics Analysis Identifies Potential Ferroptosis Key Genes in the Pathogenesis of Intracerebral Hemorrhage
Source: Front Neurosci. 2021 Jun 7;15:661663. doi: 10.3389/fnins.2021.661663 (PMC8215678; doi:10.3389/fnins.2021.661663)
Supplement: Supplementary file 1 [file Table_1.DOCX]

| **Number** | **GSE dateset** | **Title** | **Status** | **Sample** |
| --- | --- | --- | --- | --- |
| **1** | **GSE24265** | [**Expression data from human brain samples**](https://www.ncbi.nlm.nih.gov/geo/query/acc.cgi?acc=GSE24265) | **Public on Sep 24, 2010** | **mRNA in brain perihematomal tissue from ICH patients** |
| 2 | GSE163256 | [Longitudinal transcriptomics define the stages of myeloid activation in the living human brain after intracerebral hemorrhage](https://www.ncbi.nlm.nih.gov/geo/query/acc.cgi?acc=GSE163256) | Public on Feb 19, 2021 | Myeloid cells from cerebral hemorrhage patients |
| 3 | GSE138092 | [microRNA expression Data from cerebrospinal fluid of cancer patients](https://www.ncbi.nlm.nih.gov/geo/query/acc.cgi?acc=GSE138092) | Public on Sep 29, 2020 | Extracellular miRNA was extracted from cerebrospinal fluid |
| 4 | GSE134505 | [Prdx1, a RNA binding protein, regulate inflammatory injury in intracerebral hemorrhage by targeting RNA stability](https://www.ncbi.nlm.nih.gov/geo/query/acc.cgi?acc=GSE134505) | Public on Apr 10, 2020 | HeLa cells |
| 5 | GSE134504 | [Prdx1, a RNA binding protein, regulate inflammatory injury in intracerebral hemorrhage by targeting RNA stability (PRDX1_iRIP-seq)](https://www.ncbi.nlm.nih.gov/geo/query/acc.cgi?acc=GSE134504) | Public on Apr 10, 2020 | HeLa cells |
| 6 | GSE134503 | [Prdx1, a RNA binding protein, regulate inflammatory injury in intracerebral hemorrhage by targeting RNA stability (PRDX1_OE_RNA-seq)](https://www.ncbi.nlm.nih.gov/geo/query/acc.cgi?acc=GSE134503) | Public on Apr 10, 2020 | HeLa cells |
| 7 | GSE125512 | [Intracerebral Hemorrhage Induces Inflammatory Gene Expression in Peripheral Blood: Global Transcriptional Profiling in ICH Patients](https://www.ncbi.nlm.nih.gov/geo/query/acc.cgi?acc=GSE125512) | Public on Apr 21, 2019 | The peripheral blood of ICH patients |
| 8 | GSE75676 | [Identification of Circulating Fibrocytes and Dendritic Derivatives in Corneal Endothelium of Patients with Fuchs' Dystrophy](https://www.ncbi.nlm.nih.gov/geo/query/acc.cgi?acc=GSE75676) | Public on Jan 24, 2017 | Fresh corneal endothelial |

| 9 | GSE75675 | [Identification of Circulating Fibrocytes and Dendritic Derivatives in Corneal Endothelium of Patients with Fuchs' Dystrophy [RT-qPCR array CAPH10410]](https://www.ncbi.nlm.nih.gov/geo/query/acc.cgi?acc=GSE75675) | Public on Jan 24, 2017 | Fresh corneal endothelial |
| --- | --- | --- | --- | --- |
| 10 | GSE75674 | [Identification of Circulating Fibrocytes and Dendritic Derivatives in Corneal Endothelium of Patients with Fuchs' Dystrophy [RT-qPCR array CAPH10409]](https://www.ncbi.nlm.nih.gov/geo/query/acc.cgi?acc=GSE75674) | Public on Jan 24, 2017 | Fresh corneal endothelial |
| 11 | GSE67281 | [Identification of Dysregulated Long Noncoding RNAs in the Midbrain of Human Cocaine Abusers](https://www.ncbi.nlm.nih.gov/geo/query/acc.cgi?acc=GSE67281) | Public on Jul 04, 2015 | LncRNA in midbrain of chronic cocaine abusers |
| 12 | GSE43618 | [Identification of inflammation-related microRNAs deregulated in the plasma from intracerebral hemorrhage patients](https://www.ncbi.nlm.nih.gov/geo/query/acc.cgi?acc=GSE43618) | Public on Jan 19, 2013 | MicroRNAs in plasma from ICH patients |
| 13 | GSE17294 | [Molecular Remodeling of Ion Channels in Human Atrial and Ventricular Myocytes Associated with Ischemic Cardiomyopathy](https://www.ncbi.nlm.nih.gov/geo/query/acc.cgi?acc=GSE17294) | Public on Feb 18, 2010 | Ventricular myocytes from Ischemic cardiomyopathy of patients |

In these 13 datasets, only GSE24265 is human brain tissue and its mRNA expression profile.
